# Towards Better Delivery of Cannabidiol (CBD)

**DOI:** 10.3390/ph13090219

**Published:** 2020-08-28

**Authors:** Sophie Anne Millar, Ryan Francis Maguire, Andrew Stephen Yates, Saoirse Elizabeth O’Sullivan

**Affiliations:** 1 Artelo Biosciences, 888 Prospect Street, Suite 210, La Jolla, CA 92037, USA; sophieannemillar@gmail.com (S.A.M.); andy@artelobio.com (A.S.Y.); 2 Division of Graduate Entry Medicine and Medical Sciences, School of Medicine, University of Nottingham, Royal Derby Hospital, Derby DE22 3DT, UK; Ryan.Maguire@nottingham.ac.uk

**Keywords:** cannabidiol, CBD, formulation, clinical development

## Abstract

Cannabidiol (CBD) has substantial therapeutic potential, but its development as an effective drug by the pharmaceutical industry is hindered by intrinsic characteristics such as low bioavailability, low water solubility, and variable pharmacokinetic profiles. Importantly, lack of patentability of the drug substance also limits the likelihood of an expensive, full development programme in anything other than orphan indications. Potential avenues to overcome these issues with CBD include self-emulsifying drug delivery systems, improved crystal formulations and other solid-state delivery formulations, which are mostly in the pre-clinical or early clinical stages of development. This review identifies issues compromising current delivery of solid-state CBD, and how advanced pharmaceutical development strategies can enable CBD to realise the full potential as a successful therapeutic agent.

## 1. Introduction

Cannabidiol (CBD) is a phytocannabinoid used globally for a variety of indications, but with few approved medicinal applications. Purified CBD is only licensed in treatment-resistant, rare paediatric forms of epilepsy [1,2,3,4,5]. Ongoing clinical trials are being conducted in potential indications such as anxiety, schizophrenia, addiction, post-traumatic stress disorder, graft-versus-host disease, cancer and inflammatory bowel disease. The United States (US) Food and Drug Administration and the European Medicine Agency approved drug formulations containing CBD include Epidiolex^®^ (a pure CBD oral solution) and Sativex^®^ (a CBD and Delta-9-tetrahydrocannabinol (THC, 1:1) oromucosal spray), both developed by GW pharmaceuticals.

In order to be successfully utilised as a medicine, it is paramount to identify and overcome the inherent challenges that face CBD’s effective delivery, particularly through the oral route, which is the most preferred route for drug delivery by patients and drug developers. Some of the most significant issues with oral CBD include poor bioavailability, variable pharmacokinetics profiles, and possible polymorphisms [6], which may have unintended consequences of less predictable efficacy, increased side effects and drug–drug interactions with higher doses. This review will outline some of the current issues with CBD pharmaceutics, the novel CBD formulations under development and under clinical investigation, and the strategies to improve CBD delivery and efficacy.

## 2. The Problems with CBD

### 2.1. Bioavailability and Pharmacokinetics

Successful drug delivery to the intended target sites of action is dependent on multiple factors including the individual’s physiology and the drug’s physicochemical properties (solubility, dissolution, stability, permeability and metabolism). Poor bioavailability, which is dependent on these factors, generally leads to insufficient therapeutic efficacy and is more likely to produce high inter-individual variability in pharmacokinetic (PK) parameters [7,8]. In this context, the bioavailability of CBD varies greatly with route and mode of administration [9,10]. The oil/water partition coefficient (Log *P*) describes a drug preference which exists either in the water or oil part of a solution; higher Log *P* values mean more of the drug will be distributed in the oil part of the solution. Due to the highly lipophilic nature of CBD (Log *P* 6.3) [11], it is most commonly supplied as an oil or alcoholic formulation either in soft-gel capsules, liquid solution, sublingual drops, or as an oromucosal spray [12]. Studies examining oral and oromucosal delivery of CBD and THC at equimolar concentrations in humans show high inter/intra-individual variability [13,14].

Highly lipophilic drugs delivered orally in solution can precipitate in the gastrointestinal (GI) tract, resulting in an absorption rate slower than elimination [8]. The oral bioavailability of CBD is estimated at 6%, although data in this area have been noted to be considerably lacking [15]. Time to peak plasma concentration following oral delivery is slow (1–4 h), the *C*_max_ (maximum concentration) generated from 20 mg of orally delivered CBD/THC sprayed onto a gelatin capsule was 2.4 ng/mL of CBD, and the half-life of CBD was reported between 1.4 and 10.9 h after oromucosal spray [14,15]. Oil suspensions designed for oral and oromucosal routes of administration are currently favoured; Epidiolex^®^ is delivered orally in an oil solution, and Sativex as an oromucosal spray. The oromucosal route circumvents some of the problems associated with the oral route, and provides a more rapid onset of action. However, Itin and colleagues suggest that a substantial proportion of the oromucosal delivered dose may actually be absorbed through the GI tract [16]. Authors noted that the PK of Sativex is different when administered in a fasting state relative to a fed state (as is Epidiolex), but that this should not be the case as PK profiles should only be substantially influenced by a fed-state when consumed orally. Furthermore, in work published by Guy and colleagues, the PK parameters of Sativex were similar between oromucosal and oral routes of administration [14]. These observations are in agreement with further work by Itin and colleagues, demonstrating CBD’s lipophilic nature causes it to accumulate in the oral mucosal lining or enter the GI tract upon swallowing [17]. However, the authors suggest this route of administration remains viable, so long as exposure times to the oral mucosal lining are adequate, and a method of preventing washout of the drug by saliva is present.

A potential method to increase oral bioavailability is to administer CBD alongside a high-fat/high-calorie meal [16]. This has been demonstrated in healthy subjects with about four-fold increases in bioavailability compared to fasted, and in adult patients with refractory epilepsy with four- and fourteen-fold increases in area under the curve (AUC) and *C*_max_, respectively, in the fed state compared to fasted [18,19,20]. This is likely due to increased micelle and chylomicron formation making more drugs available for lymphatic transport [21,22]. High fat meals also potentially inhibit the activity of drug efflux transporters present on the apical membrane of enterocytes, and stimulate the release of biliary secretion, which further inhibits efflux transporter activity [23,24]. Although lymphatic transport bypasses the liver into systemic circulation, CBD delivered orally is still subject to first pass metabolism. One study showed the AUC of 7-hydroxy-cannabidiol (7-OH-CBD) is about 40% the level of orally delivered CBD, however participants were also receiving concomitant treatment Clobazam, Valproate or Stripentol [25]. Indeed, impaired hepatic metabolism has been shown to cause an increase in the bioavailability of CBD, and indicates first pass metabolism represents a significant barrier in increasing the bioavailability of CBD [26].

Smoking or vaporisation can overcome some of the problems of CBD delivery. The bioavailability of five individuals after smoking 20 mg of deuterium-labelled CBD was reported at 31 ± 13% (range 11–45%) [27]. Smoking allows for rapid delivery of CBD, and peak plasma levels occurred within 3 min following inhalation (110 ± 55 ng/mL). A more recent study assessing the pharmacokinetics of vaporised CBD (100 mg) in healthy men (*n* = 6) and women (*n* = 6) showed a mean *C*_max_ of 125.4 ng/mL ± 95.2 in men and 83.7 ng/mL ± 8.8 in women [28]. This study also compared vaporised CBD to orally delivered CBD at the same dose, and found vaporisation produced around a ten-fold increase in C_max_. Whilst vaporisation increases the bioavailability of CBD, large standard deviations indicate there is still substantial variability in inter-subject pharmacokinetics. Furthermore, the use of solubilisers required to create a vaporisable solution can result in irritation of the airways and may not be suitable for all patients. Smoking a cannabis-based medicinal product is also not currently allowed under legislation in the UK.

A number of alternative delivery methods by-passing the digestive tract and first pass metabolism are currently under investigation including transdermal and nasal routes, and eye drops, which have been reviewed elsewhere [29].

### 2.2. Stability

CBD can be degraded depending on temperature, light and auto-oxidation. Mazzetti and colleagues examined nine commercially available CBD E-liquids and found an average degradation by 13% in 30 days when exposed to light and room temperature. However, samples stored in the dark at room temperature only suffered 4% degradation, suggesting light is a significant contributing factor in degradation at room temperature [30]. Another recent analysis found that CBD samples stored in the dark at room temperature for three months showed the presence of Δ^9^-THC and Δ^8^-THC impurities [31]. Whether the stability of CBD products is altered by different formulations is not known [32]. However, one study demonstrated that CBD dissolved in medium-chain triglycerides (MCT) oil is completely devoid of any lipid oxidation products, indicating MCT oil matrices are less susceptible to oxidative degradation than olive oil or hemp seed oil. Oxidative processes are associated with rancidity and deterioration of fats, as well as decreased concentrations of cannabinoids and terpenes [33]. Epidiolex^®^ (suspended in sesame seed oil and ethanol) and Sativex (suspended in ethanol anhydrous, propylene glycol and peppermint oil) are said to be stable 56 and 48 days after opening the container, respectively (see prescribing information (https://www.accessdata.fda.gov/drugsatfda_docs/label/2018/210365lbl.pdf) and online website (http://sativex.co.uk/ms-nurses/frequently-asked-questions/)).

### 2.3. Dosing and Side Effects

In our systematic review, within randomised controlled trials administering CBD (by oral solution, capsules, or oromucosal spray), an average dose of 14 mg/Kg/day was reported in positive outcome trials, compared to 5 mg/Kg/day average dose reported in trials in which CBD did not demonstrate efficacy [34]. The high oral doses that are required for efficacy may impact the occurrence of adverse events (AEs), as well as increasing drug costs to the healthcare provider or patient.

CBD is generally described as well tolerated, with a favourable safety profile, and does not demonstrate any evidence of abuse potential [35,36]. CBD has been most extensively studied in the clinical setting of patients with epilepsy. Within these trials, the most common side effects observed have been somnolence/sedation, diarrhoea, loss of appetite, fatigue, and sleep disturbances [37]. Concomitant increases in anti-epileptic drug metabolites because of drug–drug interactions (DDIs) with CBD may contribute to particular side effects observed such as somnolence [2,38]. A recent review which analysed prescribing information and new drug applications from federal agency websites (U.S. FDA, Health Canada, and others) reported that nearly one half of CBD users experienced an AE, which generally followed a dose-dependent relationship [39]. In one randomised controlled trial in treatment resistant epilepsy, AEs were reported in 93% of patients taking CBD and included vomiting and diarrhoea, while 86% of patients in another trial reported similar AEs [4,40]. A recent systematic review and meta-analysis on the clinical benefits of CBD found mild side effects to be present in 76% of patients taking purified CBD (such as Epidiolex^®^) and 33% in those taking CBD-rich extracts, and this was statistically different from placebo [41]. It was also noted that those receiving pure CBD reported administering higher doses compared to those taking CBD-rich extracts (25 mg/Kg/day vs. 6 mg/Kg/day), without differences in efficacy. It is unclear whether CBD-rich extracts demonstrate a more favourable side effect profile due to the presence of non-standardised herbal compositions, improved absorption of the CBD in the more complex mixture of compounds or whether it is due to the lower doses of individual cannabinoids reportedly consumed. It is also noteworthy that all CBD-rich extract studies were retrospective records or online surveys, while pure CBD trials were prospective, randomised controlled trials [41]. Thus, it is not clear whether various CBD formulations have different side effect profiles.

### 2.4. Drug–Drug Interactions

Drug–drug interactions (DDIs) with CBD are a high risk as it is metabolised by, and a competitive inhibitor of, CYP450 enzymes (specifically CYP3A4 and CYP2C19) [39,42]. A case report of a 32-year-old woman with refractory epilepsy receiving tacrolimus (5 mg/day) and CBD (2000–2900 mg/day) reported that after two weeks of co-administration there was a threefold increase in dose-normalised tacrolimus concentrations [43]. A case report of a 37-year-old male similarly suggested CBD can increase warfarin concentrations [44]. More robust evidence of DDIs comes from a meta-analysis assessing four randomised, double-blind, placebo-controlled trials in Lennox-Gastaut syndrome (LGS) and Dravet syndrome (DS) (*n* = 396 LGS, 318 DS) [45]. Authors noted incidences of serious AEs were 8% higher in CBD groups relative to placebo, and in patients taking clobazam alongside CBD, AEs were 14% more common than CBD alone. Blood plasma levels of the pharmacological active metabolite of clobazam, *N*-desmethylclobazam, were three times higher when administered alongside CBD. Likewise, increases in the CBD metabolite, 7-OH-CBD, are also seen when these drugs are co-administered [46]. Valprorate, another common anti-epileptic drug, is strongly associated with increased liver transaminases levels with CBD use. An FDA report describing clinical data for Epidiolex^®^ in the treatment of LGS or Dravet syndrome highlighted alanine aminotransferase (ALT) levels were three times the normal limit in 13% of Epidiolex^®^ treated patients. This AE was also true for patients taking clobazam, but was more common in valproate treated patients. Another study investigating the potential for DDIs with cannabis-based medications using the FDA drug interaction database identified three of the main cytochrome enzymes (CYP3A4, CYP2C9 and CYP2C19) responsible for 20–70% of total cytochrome p450 activity were inhibited by CBD [47]. Furthermore, following oxidative reactions at phase 1 metabolism, CBD is then subject to phase II glucuronidation reactions by the enzymes UGT1A9 UGT2B7 and UGT1A7. Competitive binding for these enzymes between drugs also presents another point at which drug metabolite levels could be altered [47]. These observations indicate the potential for DDIs with CBD in a variety of conditions are likely, and needs to be investigated using large patient studies.

### 2.5. Polymorphisms

In material science, a polymorphism refers to the occurrence of differing crystalline structures of the same chemical compound which can be due to the crystallisation conditions (such as the rate of crystallisation) leading to different molecular conformations [48]. Polymorphic substances have identical chemical composition, thus will demonstrate the same chemical behaviour once in solution. Polymorphism screening is a vital component of pharmaceutical drug development as the existence of polymorphisms can affect drug performance and characteristics; for example, a fourfold difference in solubility can occur between different forms due to differences in dissolution rates [49]. An active pharmaceutical ingredient (API) based on a single polymorphism is likely to have an improved and consistent safety and efficacy profile. These properties may include thermodynamic, kinetic, surface, and mechanical properties, amongst others [48]. Polymorphism is acknowledged as an issue in drug development by key regulatory agencies who have issued guidance on approaches used to control for and to justify using polymorphic forms in drug substances. As stated by the FDA’s guidance document “polymorphism can affect the quality, safety, and efficacy of the drug product”; so controlling polymorphism from the drug substance can provide an easier development with regards to regulatory approval [50]. CBD has been shown to be present as two or more inherent crystalline forms which have potential effects on drug absorption and bioavailability due to different physicochemical properties [6]. To date, no studies have assessed how different crystalline forms may impact the pharmacokinetic profile of CBD, which may have implications for CBD being developed as solid-state forms.

## 3. Novel CBD Medical Products in Development

Due to the challenges with CBD, many companies are trying to develop CBD medicines with better PK profiles. These strategies include self-emulsifying drug delivery systems, improved single crystal structures, and CBD cocrystals. Table 1 summarises some of the different CBD formulations under development by commercial companies with information in the public domain.

### 3.1. Self-Emulsifying Drug Delivery Systems

Methods to increase oral CBD bioavailability have included self-emulsifying drug delivery systems (SEDDS). These involve mixtures of oils, surfactants, and solvents that produce nano or micro sized droplets when they come into contact with an aqueous solution such as in the gut [51]. The small nature of the droplets increases the surface area available for drugs to be dissolved and absorbed. For example, soft gelatin capsules containing CBD developed by Satipharm, based on an advanced self-nanoemulsifying technology, have demonstrated greater bioavailability (about 31–34% higher compared to a reference oromucosal spray), solubility, and faster time to peak plasma concentrations in humans [52,53,54]. However, inter-individual variations were still high. This formulation has been proven safe and effective in a paediatric population with treatment-resistant epilepsy [1]. Another group recently reported a 4.4-fold increase in C_max_, enhanced bioavailability, and shorter *T*_max_ with a SEDDS-CBD formulation based on VESIsorb^®^ technology compared to a control CBD oil [51]. VESIsorb^®^ is a novel lipid-based delivery system which self-assembles on contact with an aqueous phase into a colloidal delivery system, which solubilises the drug and improves diffusion and absorption and thus bioavailability [55]. In rats, another approach of a nano-emulsion (NE) formulation has been tested. CBD-NE oil drops increased absorption and bioavailability (21% higher) compared to a control CBD oil [56]. However, there may be practical (stability, complex formulations, safety of excipients, scalability) and economic (cost of formulation, patentability) issues with this method of increasing oral CBD bioavailability.

Although the methodology is not clear from publicly available information, Echo Pharmaceuticals and Ananda Scientific are also investigating formulations which claim to enhance bioavailability and consistency in PK profiles by increasing CBD’s water solubility; Ananda’s Liquid Structure™ Enhanced CBD and Echo Pharmaceutical’s Arvisol, using their lipophilic compound delivery technology Alitra^®^. Both compounds are in preclinical or early clinical phase 1 development (see Table 1).

Another encapsulated form of CBD is APH-1501 (produced by Aphios), which are time-released capsules in which CBD is encapsulated in biodegradable polymer nanospheres as a lyophilised powder. This CBD is awaiting phase 2 testing in opioid addiction.

### 3.2. Solid-State Delivery Formulations

Solid-state oral delivery allows for 100% of the drug to reach the GI tract and has the potential to improve PK characterisation [76,77]. CBD delivered via this route would also further avoid local side effects associated with use of Sativex oromucosal spray (1:1 CBD:THC) or GI discomfort or pain associated with the vehicle itself in oral liquid formulations [78]. Current investigated solid-dose oral formulations of CBD include a 200 mg CBD tablet by Columbia Care called BeneCeed™, which will be used in a UK clinical trial. Elsewhere, a patent by GW pharmaceuticals lists a solid-state CBD as a potential clinical consideration in the treatment of inflammatory bowel disease [79]. Whilst dosing in this fashion ensures a consistent dose, formulations of this nature do not necessarily address problems associated with poor bioavailability.

### 3.3. Improved Single Crystal Structures

Some researchers claim to have improved the single crystal form of CBD. For example, one patent listed describes a crystalline CBD of a novel form, including (*R*,*R*)-(−)-crystalline cannabidiol [80]. This crystalline form was shown to possess a melting point of 37–50 °C, compared with a melting point of 66–67 °C for CBD. Intramolecular crystal lattice binding between ions within a crystal affects its melting point and reductions in lattice energies may increase aqueous solubility [8]. PureForm CBD™ is described as a molecularly identical, non-hemp-based CBD that has been developed using their Inter-Molecular Stacking Technology to improve solubility and stability [81]. There is no further publicly available information on these products.

### 3.4. Cocrystal Engineering as a Potential Solution for CBD Oral Delivery

Interest and progress in the concept of cocrystallisation have expanded over recent years and is becoming a well-established process in drug development. Cocrystals consist of the API and one or more unique crystalline co-formers which modify the material properties whilst retaining the intrinsic pharmacological drug activity. Cocrystallisation is a useful method for overcoming problematic properties of drugs by increasing the bioavailability, solubility, dissolution rate, physical form, melting point, tableting, stability, or permeability of drug substances [82,83,84]. Further advantages of crystal preparations include the potential for numerous co-molecules including preservatives, other APIs, and pharmaceutical excipients, as well as providing the opportunity to address intellectual property issues by extending API life cycles and fulfilling patent eligibility criteria [85].

Entresto™ is an example of a drug–drug cocrystal containing monosodium sacubitril and disodium valsartan used to treat chronic heart failure that has obtained FDA approval. PK studies demonstrated a mean relative bioavailability of 161% in the cocrystal form of valsartan compared to reference valsartan tablets [86]. The cocrystal demonstrates high solubility and medium permeability. Suglat^®^ is another marketed cocrystal, comprised of the sodium glucose cotransporter 2 (SGLT2) inhibitor ipragliflozin and L-proline, approved in Japan for the treatment of diabetes mellitus. The third cocrystal currently on the market is Depakote^®^, an anti-convulsant drug, which is comprised of valproate sodium with valproic acid [87].

Artelo Biosciences have developed a cocrystal with CBD that was designed to take advantage of cocrystal properties and help alleviate some of the problems with CBD delivery. This cocrystal uses the co-former tetramethylpyrazine (TMP; also called ligustrazine), a plant-derived compound from the Ligusticum species that is widely used in Chinese medicine. TMP may offer increased efficacy and bioavailability, by acting synergistically and changing the physiochemical properties that are associated with ineffective absorption. ART12.11 (CBD:TMP cocrystal) is currently in the nonclinical phase of pharmaceutical development targeted towards post-traumatic stress disorder (PTSD), inflammatory bowel disease (IBD), stroke and rare diseases, and has been recently granted a composition of matter patent in the US.

### 3.5. Other Delivery Systems and Formulation in Development

An oral capsule developed by Lexaria Bioscience Corp called “TurboCBD” claims to result in increased circulating CBD levels compared to control CBD, and contains American ginseng, ginkgo biloba, and organic hemp oil, produced using DehydraTECH™ delivery technology [69].

Preveceutical’s “Sol-Gel” is exploring an intranasal CBD formulation to increase bioavailability and is currently in the pre-clinical stage (see Table 1). Zynerba Pharmaceuticals have progressed a permeation-enhanced CBD gel “Zygel” for transdermal application to phase 2 trials [75]. Botanix pharmaceuticals are exploring a number of gel formulations for transdermal application in indications such as acne, psoriasis and dermatitis that are in early clinical development.

Kalytera are also exploring inflammatory skin conditions using an L-valine-ester derivative of CBD for topical delivery, which is in pre-clinical stages. Kalytera are also developing a bi-sulphate derivative of CBD for oral delivery which claims to be water soluble, a bi-phosphate CBD derivative aimed for intra-tracheal delivery via a novel aerosolised formulation, and an intravenous (IV) formulation (see Table 1). GW Pharmaceuticals list an IV formulation in phase 1 trial for neonatal hypoxic-ischemic encephalopathy (NHIE).

A sublingual formulation by Diverse Biotech Inc., and an oral liquid by Emerald Health Pharmaceuticals containing a pure synthetic CBD are both in early clinical phases (see Table 1).

Complexation of CBD with cyclodextrins (CD) has also been investigated as a potential method to increase the water solubility and subsequently improve the bioavailability of sublingually delivered CBD. Mannila and colleagues demonstrated precipitation complexation of CBD and β-CD at a 1:2 ratio could increase the water solubility of CBD and increase the dissolution rate [88]. The authors noted sublingual delivery of the CBD/β-CD complex produced superior bioavailability compared to oral dosage forms of CBD in rabbits. However, in this study, CBD delivered in an ethanol solution sublingually was comparable to sublingual delivery of the CBD/β-CD complex. Two formulations of CBD and CDs are currently in development by Medexus pharmaceuticals and Vireo health LLC. These companies propose complexes of CBD and CDs will increase the aqueous solubility and subsequently improve bioavailability. However no clinical studies have been performed using these exact formulations to date.

## 4. Conclusions

Intrinsic problematic issues, such as polymorphism, low aqueous solubility and poor bioavailability, hinder the effective clinical development of CBD as an oral drug in the solid-state. SEDDS offer a way to increase bioavailability by increasing the solubilisation of CBD in the aqueous environment of the GI tract, allowing more CBD to cross the unstirred water layer of the GI tract and move into lymphatic or blood circulation [51]. However, SEDDS can cause chemical instabilities of drugs and high surfactant concentrations have the potential to irritate the GI tract. Furthermore, some of hydrophilic components can diffuse into the shells of gelatine capsules causing precipitation and poor dissolution rates of the drug in the GI tract [89]. While SEDDS formulations may have merit, these approaches suffer from using a lipophilic molecule which is not optimised for absorption within the gut, and intellectual property protecting these innovations can often be circumnavigated by experienced scientists. A cocrystal modification has the potential to overcome numerous physiochemical issues, allowing improved stability and bioavailability. Cocrystals can be “fine-tuned” using various inert or pharmacologically active co-formers, which may provide a more predictable pharmacokinetic profile and subsequently reduce side effects associated with high intra- and inter-personal variability. Importantly, because co-crystal formulations offer a unique and defined composition of matter, patentability ensures financial investment is available to push CBD from bench to bedside. What is clear is that the field of cannabinoid-based medicines, with CBD as a leading example, has a rich and diverse future ahead with continued preclinical research and a strong clinical pipeline. If companies are able to overcome the challenges facing the druggability of CBD, or indeed other cannabinoids or phytochemicals, in the oral solid dosage form, and protect their investment through strong intellectual property, it will undoubtedly lead to the best chance of wide therapeutic applications, offering solutions to many more patients.

## Figures and Tables

**Table 1:** Cannabidiol (CBD) formulations under development by commercial companies (presented in alphabetical order) with information in the public domain. Abbreviations: CBD, cannabidiol; i.v., intravenous; N/A, not available; NHIE, Neonatal Hypoxic-Ischemic Encephalopathy; TMP, tetramethylpyrazine; PPARγ, peroxisome proliferator-activated receptor gamma; CB2, cannabinoid type 2 receptor; US, ultrasound; BBB, blood–brain barrier.

Company	Name	Formulation (If Available)	Delivery	PK Data/Other	Clinical Development	References
Ananda Scientific	Enhanced CBD	ANANDA’s Liquid Structure™ Enhanced CBD	N/A (presumed oral)	Reported as: fully water soluble (100%); high bioavailability (20× more than other CBD oils at 30 min); no degradation in stomach; stable—storable at room temperature for at least 2 years; consistent dosing—reliable results with use	Phase 1 studies underway for indications: neuropathic pain and opioid replacement and withdrawal	[57]
Aphios	APH-1501	Nanoencapsulated Cannabidiol Time Released Capsules; CBD encapsulated in biodegradable polymer nanospheres as a lyophilised powder	Oral capsule	N/A	Phase 2 trial planned for the treatment of opioid addiction	[58]
Artelo Biosciences	ART12.11	Co-crystal solid state formulation with TMP	Oral	N/A	Pre-clinical development for PTSD, IBD, Stroke and Rare Diseases	[59]
Botanix Pharmaceuticals	BTX1503	Gel	Transdermal	N/A	Phase 1b study completed, phase 2 study underway in moderate to severe acne	[60]
	BTX1308	Gel	Transdermal	N/A	Phase 1b study planned in Plaque psoriasis	[61]
	BTX1204	Gel	Transdermal	N/A	Phase 1b study—unknown status. Phase 2 study recruiting. Indication: atopic dermatitis	[62]
	BTX 1701	Facial cleanser utilising Permetrex™ skin delivery technology in conjunction with a novel oil clearing agent	Facial cleanser	N/A	Small clinical study performed in mild acne	[61]
Columbia Care	BeneCeed	200 mg CBD tablet	Oral	N/A	N/A	[63]
Diverse Biotech Inc.	BRCX014	Sublingual formulation	Sublingual	N/A	Two registered phase 1/2 trials in Cancer	[64]
Echo Pharmaceuticals	Arvisol	Lipophilic compound delivery technology Alitra^®^	Oral	Claims to improve bioavailability and steadier temporal profile	Preclinical – indicated for Rett Syndrome—Epilepsy, and Schziophrenia	[65]
Emerald Health Pharmaceuticals	EHP-101	Synthetic CBD designed to enhance the therapeutic benefits of CBD by being a dual PPARγ and CB_2_ agonist	Oral liquid	N/A	Phase 1 trial completed in healthy volunteers but no results published yet. Phase 2a trial registered but not yet recruiting for Diffuse Cutaneous Systemic Sclerosis	[66]
GW Pharmaceuticals	GWP42003	NA	i.v.	N/A	Phase 1 trial completed in NHIE	[67]
Kalytera	K-1052	CBD +(*S*)-2-amino-(1-iminoethylamino)-5-thioheptanoic acid (iNOS inhibitor) coupled pro-drug molecule	i.v.	N/A	In vivo efficacy studies in rodents completed in Sepsis-induced Acute Renal Failure and Traumatic Brain Injury	[68]
	KAL-1816	CBD conjugated with naproxen	Oral and i.v.	N/A	Patents filed in acute and chronic pain	[68]
	K-1022	Bi-sulphate derivative of CBD	Oral	Water soluble	In vivo efficacy studies performed in Ulcerative Colitis; currently ADME/PK analysis in rats, as well as non-clinical safety assessment of K-1022 in rats and dogs	[68]
	K-1012	Bi-phosphate derivative of CBD; novel aerosolised formulation	Intra-tracheally	Soluble in aqueous solution; expected to increase the bioavailability	In vivo efficacy studies carried out in rodents in Adult Respiratory Distress Syndrome	[68]
	K-1032	L-valine-ester derivative of CBD	Topical delivery	N/A	In vivo efficacy models in rodents conducted in chronic inflammatory skin diseases	[68]
Lexaria Bioscience Corp	TurboCBD	Capsules also containing American ginseng, ginkgo biloba, and organic hemp oil using DehydraTECH™ delivery technology	Oral	Increased circulating CBD levels compared to control CBD (+86% at 90 min)	On the market (over the counter)	[69,70]
Medexus Pharmaceuticals	CA2476833C	Complex of RM-β-, DM-β and TM-β cyclodextrin with CBD	N/A	Improve aqueous solubility, dissolution rate, absorption and bioavailability	N/A	[71]
Preveceutical	Sol-Gel	CBD gel	Intranasal	Expected to increase bioavailability	Pre-clinical evidence to suggest better distribution along the olfactory epithelia	[72]
PureForm Global	PureForm CBD	Molecularly identical, non-hemp-based CBD, developed using InterMolecular Stacking Technology	Liquid and powder	Expected to improve solubility and stability	N/A	[73]
Satipharm	Gelpell (PLT-101)	CBD in gelatine beads encapsulated in gastro-resistant capsules	Oral	30% higher bioavailability compared to oromucosal spray.	Phase 1 and phase 2 (efficacy in paediatric epilepsy) trials completed	[1,52]
Vireo Health LLC	US2019030170	CBD and a sulfoalkyl ether cyclodextrin	N/A	Increase water solubility	N/A	[74]
Zynerba Pharmaceuticals	Zygel^TM^ ZYN002	Permeation-enhanced gel	Transdermal	N/A	Pre-clinical, phase 1 and phase 2 trials all underway in Fragile X syndrome and other rare neuro-psychiatric conditions	[75]
